# A Comparative Study on the Suitability of Smartphones and IMU for Mobile, Unsupervised Energy Expenditure Calculi

**DOI:** 10.3390/s150818270

**Published:** 2015-07-27

**Authors:** Angel Ruiz-Zafra, Eva Orantes-González, Manuel Noguera, Kawtar Benghazi, Jose Heredia-Jimenez

**Affiliations:** University of Granada, Avda. del Hospicio, Granada 18071, Spain; E-Mails: maevor@ugr.es (E.O.-G.); mnoguera@ugr.es (M.N.); benghazi@ugr.es (K.B.); herediaj@ugr.es (J.H.-J.)

**Keywords:** energy expenditure, accelerometers, physical activity, metabolic equivalent of task, smartphones, mobile, MET

## Abstract

The metabolic equivalent of task (MET) is currently the most used indicator for measuring the energy expenditure (EE) of a physical activity (PA) and has become an important measure for determining and supervising a person’s state of health. The use of new devices which are capable of measuring inertial movements by means of built-in accelerometers enable the PA to be measured objectively on the basis of the reckoning of “counts”. These devices are also known as inertial measurement units (IMUs) and each count is an aggregated value indicating the intensity of a movement and can be used in conjunction with other parameters to determine the MET rate of a particular physical activity and thus it’s associated EE. Various types of inertial devices currently exist that enable count calculus and physical activity to be monitored. The advent of mobile devices, such as smartphones, with empowered computation capabilities and integrated inertial sensors, has enabled EE to be measure in a distributed, ubiquitous and natural way, thereby overcoming the reluctance of users and practitioners associated with in-lab studies. From the point of view of the process analysis and infrastructure needed to manage data from inertial devices, there are also various differences in count computing: extra devices are required, out-of-device processing, etc. This paper presents a study to discover whether the estimation of energy expenditure is dependent on the accelerometer of the device used in measurements and to discover the suitability of each device for performing certain physical activities. In order to achieve this objective, we have conducted several experiments with different subjects on the basis of the performance of various daily activities with different smartphones and IMUs.

## 1. Introduction

In today’s world, an ever-increasing emphasis is being placed on health and wellness. People are becoming more aware of the importance of physical activity (PA) both in terms of its ensuing positive health benefits and also its contribution to successful aging by improving and maintaining health [1,2,3]. For these reasons, the World Health Organization (WHO) recommends PA for children, adults and the elderly for improving cardiorespiratory and muscular fitness, bone health, and cardiovascular and metabolic health biomarkers.

One concept which is closely related to PA and its intensity is energy expenditure (EE) and EE is commonly determined by measuring the metabolic equivalent of task (MET) [4]. The MET was conceived to provide a homogeneous estimation of the EE that an activity would involve for anyone performing it, regardless of their physiological characteristics (e.g., weight, height, *etc.*). One MET is equivalent to 3.5 mL·kg^−1^ min^−1^ and represents a person’s energy expenditure while seated and idle. This unit of measurement offers an alternative EE estimation to traditional activity tables and (approximate) calories, which may differ from one individual to another. For example, while two seated people consume the same number of METs (one MET per time unit in this case), each expends a different number of calories according to their physiology and activity duration. Much work has been published to analyze the equivalence between the execution of a certain PA and the number of expended calories associated to that activity [5,6].

It is important for EE to be measured accurately in order to understand the prevalence of meeting physical activity recommendations, identify populations and understand the relationship between physical activity and health [7]. Traditionally, physical activity has generally been assessed with self-report methods such as questionnaires where participants record information about the activities they performed [8,9]. However, the data obtained from these approaches is subjective and could be influenced by poor compliance, poor memory and cognition health status [8]. In recent years, such limitations have promoted the use of accelerometers as a substitute for self-reporting methods for an objective PA assessment [2,10].

Accelerometers satisfy many of the requirements for PA assessment, such as the possibility of measuring it in free-living conditions with minimal discomfort for the subject and in a representative time frame for the average activity level [11]. Such devices also offer a number of desirable features in monitoring human movement in general, such as adaptability to frequency and intensity of human movements, enhancements in the technology of micro-electromechanical systems (MEMS) that provide low cost, miniaturized accelerometers and also demonstrate a high degree of reliability [12].

The use of accelerometers also represents a versatile alternative to questionnaire-based methods for estimating EE, enabling measurement both indoors and outdoors [13]. In this study, we use the concept of count [14] to assess physical activity. Unlike the MET, for which there is an equivalent number of calories [6], there is no consensus or standard calculation (in calories or METs) about how counts are to be computed and the time baseline to be used (e.g., counts per minute). The number of PA counts is therefore dependent on the device features and calculation process used.

In this study, different accelerometers were tested to analyze their capabilities for estimating the EE of a series of everyday activities. The goal was to provide further insights into the data they supply, measurement accuracy, the collection process, the activities for which they could be used and the need for additional hardware to interact with them in order to obtain a larger picture about their suitability for conducting EE studies. Our aim was also to gain experience of the use of these devices from the standpoint of the comfort of the monitoring process and the feasibility of undertaking EE studies using mobile devices. For this aim, five subjects performed various everyday activities. Each activity was carried out and supported using five different accelerometers: three smartphones and two external IMU devices. With the results obtained, we shall discuss how device features (e.g., sampling frequency and acceleration sensitivity) affect EE estimation.

The paper is organized as follows: Section 2 discusses related work, Section 3 provides a brief background to energy expenditure; Section 4 introduces the study: experiments conducted and discusses the results obtained are presented in Section 5; and finally Section 6 outlines the conclusions of the study.

## 2. Related Work

The calculation of energy expenditure (EE) is by no means an easy task and various works have been published in recent decades that aim to provide a good method of estimating it. Traditionally, EE was calculated on the basis of medical features such as the basal metabolic rate (BMR) that allowed a close EE approximation to be obtained by generalizing the results (regardless physiological information) [15]. More recently, the use of methods such as indirect calorimetry or bioelectrical impedance have also been used to provide an approximation of the empirical EE [16].

Recent proposals propose measuring physical activity (PA) using accelerometers, showing the effectiveness and power of these devices for inertial force measurement [17]. Since a large number of research projects focus on different groups of users, e.g. the elderly, it is important to be able to measure EE accurately [18]. The research presented in [19,20], for example, shows how EE evolves over a period of time, determining the importance of measuring EE to enhance, or at least maintain, the user’s health and wellbeing. However, since these proposals focus on the EE of a particular PA or are based on the use of a single accelerometer [8,21,22], the results obtained are fully conditional on the technology used or by the PA performed which means that in some cases EE has a higher margin of error. In this paper, we address this issue. Various activities have been performed with different accelerometers to study how accelerometer features (e.g., sampling frequency and acceleration sensitivity) affect EE estimation.

## 3. Energy Expenditure Assessment

Energy expenditure (EE) is a key indicator that can be used to determine the intensity of a physical activity (PA). The calculation process, however, is by no means a single or closed process. Different methodologies have been proposed for this purpose [16]. As explained above, one of the most common current approaches followed to measure the PA is based on the use of accelerometers. The use of these devices enables the amount of the PA to be measured through the use of counts [23]. In conjunction with other physiological information, these counts enable an EE estimation for an activity to be calculated.

Various methods for calculating the counts from the raw accelerometer information (*i.e*., roll, yaw and pitch angles x, z, y) have been proposed and the three most used approaches [14] are as follows:

- The use of digital counters to accrue the number of times the signal crosses a preset threshold.
- The use of an algorithm to determine the maximum value for a selected period of time.
- The use of the area under the curve (integration) algorithm.

According to previous literature [14], it is the third of these which is the most scientifically popular and the one chosen for this research work.

## 4. Experiment Description

In this section, we shall first briefly describe the results of the original experimentation that gave rise to our current research [24]. We shall then explain the experiment conducted for this work in order to address some of the limitations of the first study.

### 4.1. Previous/Original Experimentation

In our previous research [24], we conducted a single case study which used three different accelerometers: two stand-alone sensor devices with open APIs and one smartphone accelerometer. We conducted 27 measurement experiments with the accelerometers in different places (on the chest or leg, or in a pocket) and three different activities were performed (watching TV, sweeping the floor and walking at 4 km/h). These activities were selected because they involve different levels of PA intensity and represent daily activities. The results demonstrated that the smartphone accelerometer, regardless of the activity being performed and its location on the body, provided the best results in terms of accuracy for energy expenditure calculation (in comparison with reference data sources and studies [5]).

### 4.2. Current Experiment

Our intention was to reinforce the conclusions drawn from our original research by broadening the study with a larger number of subjects and smartphones. We therefore decided to conduct all the experiments with the accelerometers in the same place (e.g., on the hip) for ergonomic and usability reasons. According to [25] the hip or waist is the most common place to wear an accelerometer (people in their daily lives do not usually wear devices on other parts of the body, such as on their leg or chest). We chose the hip because it is nearest the body’s center of mass in order to approximate whole body movement and energy expenditure [22].

In this research we analyzed five different types of accelerometers (two open stand-alone sensors and three smartphone accelerometers) placed on the hip for three physical activities, studying the data collection process and infrastructure required in terms of additional devices and software.

The main objectives of this study were:

- To design an experiment taking into account reasonable precautions, such as the simultaneous synchronization and measurement from each device in order to avoid any kind of bias or noise that could lead to erroneous conclusions;
- To perform several types of activities using five different accelerometers to measure the EE of these physical activities;
- To discuss the results, in order to find out whether there are any situations/contexts where it is appropriate to use one accelerometer or another depending on the PA;
- To determine which features of the accelerometers account for differences in the estimation of the EE of a series of daily life activities.

### 4.3. Energy Expenditure Estimation Procedure

As previously mentioned, EE estimation stems from the calculation of metabolic equivalent tasks (METs). The number of METs can be calculated according to the number of counts obtained from the accelerometers and other user characteristics, such as weight, height, age and gender.

**Figure 1:**
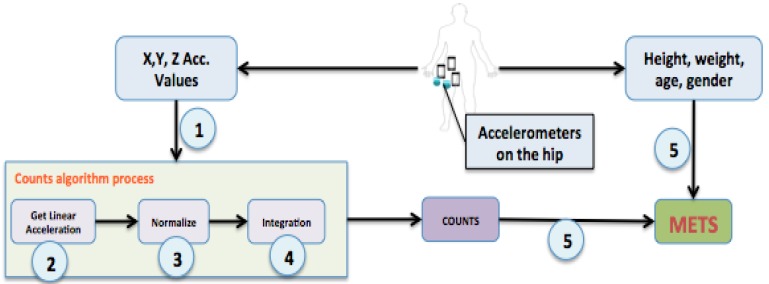
Energy expenditure estimation procedure.

Figure 1 shows the complete procedure followed in our experiment to obtain an EE estimation in MET units. The count calculation process is independent of the type of user, accelerometer features or physical activity, unlike the final MET calculation which is dependent on the user’s physiology. The following calculation procedure, numbered from 1 to 5 in Figure 1, is used to obtain the number of counts:

1. Obtain the x, y, z axis values from the accelerometer (raw data) for a period of time, *i.e.*, the duration of the physical activity.
2. There are many accelerometers on the market with values ranging from ±2 g, to ±16 g, through ±4 g, ±8 g, *etc.* in idle status, where g is the gravitational acceleration of an object on Earth. It is therefore necessary to filter the data obtained from each accelerometer so that the measures given can be normalized and this is a usual way of proceeding in research literature [22]. In particular, we applied a low-pass filter to isolate gravitational acceleration and a high-pass filter to remove gravity and obtain linear acceleration
  alpha = 0.8;
  //low-pass filter
  gravity_x = alpha * gravity_x + (1 – alpha) * x_value;
  gravity_y = alpha * gravity_y + (1 – alpha) * y_value;
  gravity_z = alpha * gravity_z + (1 – alpha) * z_value;
  //high-pass filter
  linear_x = x_value – gravity_x;
  linear_y = y_value – gravity_y;
  linear_z = z_value – gravity_z;
3. The linear acceleration values obtained in the previous step are normalized to obtain a representative positive value for each data recorded by the accelerometer as the values may range from negative to positive:
  (1) $$\sqrt{{\left(linear\_x\right)}^{2}+{\left(linear\_y\right)}^{2}+{\left(linear\_z\right)}^{2}}$$
4. We apply an integration process to calculate the area under the curve (AUC). We choose to use the trapezoidal rule [26]:
  (2) $$\overset{b}{\underset{a}{\int }}f\left(x\right)\;dx=\left(b-a\right)\times \;\frac{f\left(a\right)+f\left(b\right)}{2}$$
  The sums of these areas (raw counts) equal the total number of counts obtained by the accelerometer in the PA performed [23].
5. Although the number of counts represents the amount of physical activity, in order to estimate the EE, other physiological user values such as height, weight, gender and age are considered. We used previously validated formulas [27] to obtain a good EE estimation (in METs units) from the number of counts and physiological information.

### 4.4. Study Activities, Subjects and Devices

In this study, the sensors devices were placed inside the pocket of a waist carrier belt, with the belt pocket hanging over the front of each user’s dominant hip. The belt was tightened around the waist so as to allow free body movement. Table 1 summarizes the study parameters.

**Figure 2:**
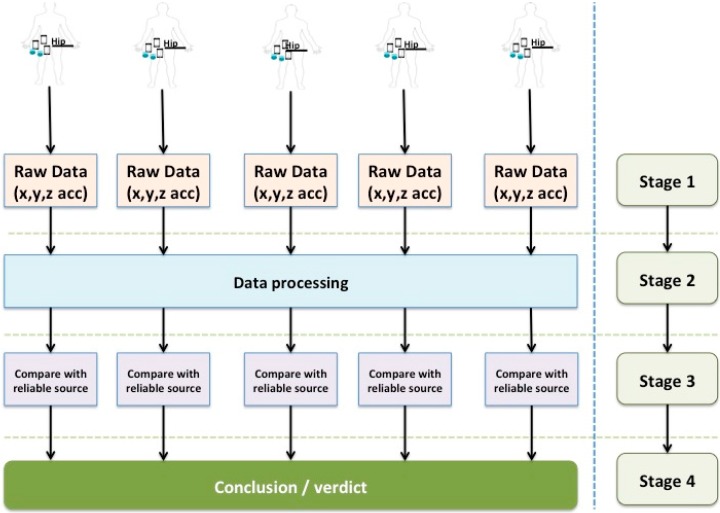
Study stages.

**Figure 3:**
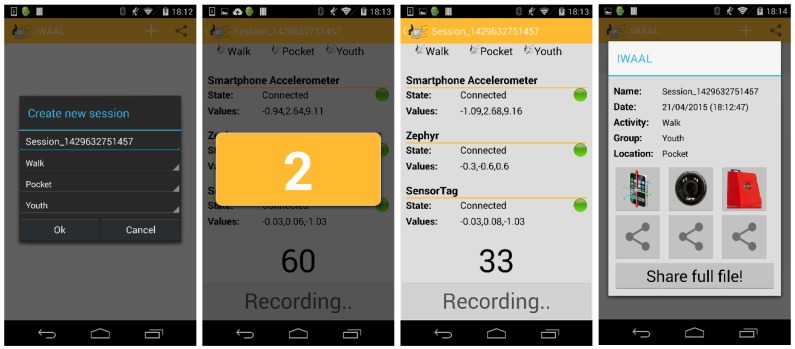
Android application developed to capture data from accelerometers.

In order to evaluate the subject and process the information obtained in each experiment correctly and objectively, we developed an Android application (see Figure 3) that implemented the count algorithm process (Figure 1). The study was further organized into four stages that are represented in Figure 2.

**Table 1:** Summary of the study features.

Subjects	Five Subjects: 1 Female: 20–40 Years Old 4 Males: 20–40 Years Old
Location(s)	Hip
Activities	Sweeping the floor Watching TV or Using the computer Walking at 4 km/h (treadmill)
Duration of the activities	60 s
Experiment schedule	All subjects performed the same physical activities in the same order: Watching TV Sweeping the floor Walking at 4 km/h
Sensors	Five sensors: Smartphone 1 (Google Nexus): 15 Hz (−16 to 16 g) Smartphone 2 (Samsung Galaxy S3): 5 Hz (−16 to 16 g) Smartphone 3 (LG L9): 20 Hz (−16 to 16 g) Bluetooth Sensor 1 (SensorTag): 1 Hz (−2 to 2 g) Bluetooth Sensor 2 (Zephyr Bioharness 3): 50 Hz (−16 to 16 g)

#### **Stage 1: “In-the-Wild” Study** 

We first arranged various sessions for the subjects to perform the physical activities. During these sessions, using the Android application specifically developed for this experiment, each subject performed each of the three physical activities with each of the three smartphones and the two external sensors, all of which were placed on the hip.

The Android application ran simultaneously on the three smartphones in order to obtain the data from each smartphone accelerometer (see Figure 3). The application was carefully designed and customized so as to synchronize and enable concurrent recording of data that each sensor/accelerometer provided simultaneously. In this way, the execution of the same activity by the same person was stored every time by each sensor in order to avoid any data bias, such as that of one person performing the same activity in a different way in subsequent stages. For example, someone might not sweep the floor in the same way the first time as they did the fifth (since we were testing five accelerometers). This also indirectly certainly made the experiment less of a chore as sweeping the floor the first time is not the same as sweeping it the fourth, for example. In addition, the two external accelerometers were connected to a single smartphone. The computational power of the smartphone allows data from several accelerometers to be recorded without affecting the performance of other tasks. The third picture in Figure 3 shows a snapshot of the Android application recording data from two sensors.

Once each subject had completed the three activities, the collected data was sent by email via the Android application to centralize the information for subsequent processing (third picture in Figure 3), specifying the group (young person, adult, elderly person), the activity performed, the session number, and the device on which the files had been collected (smartphone) and that the application was running. Each subject performed the three activities with the five different devices, and a total of seventy-five files were generated for processing.

#### **Stage 2: Data Processing** 

Once all the subjects had completed the sessions and the information had been sent, we processed the raw data recorded by each subject participating in the experiment. The raw data was processed using another custom software (desktop software implemented for this purpose), which read the files (csv format), parsed the information and returned:

- the total number of values in each file (a file represents the values from one sensor in one physical activity for 60 s).
- the number of counts applying the algorithm procedure presented in Figure 1, using a fixed interval with two values. This variable should be adjusted according to the features of the devices and type of activities.
- the number of METs using the research formulas [27].

This stage is explained in Section 5 (Results).

#### **Stage 3: Results Review** 

Using the number of METs provided by each accelerometer in each activity and by each user, we generated different charts in order to clarify the results. The proper dissemination of the information enables us to understand how the various features of the different devices provide different results and the correlation between these. These results will be presented in Section 5 (Results).

#### **Stage 4: Conclusions/Verdict** 

In the final stage we decide which device features affect EE estimation and the relation between these features according to device and EE obtained. This stage corresponds to Section 4 (Discussion) of this paper. It is important to highlight that in order to conduct the study correctly, several issues were addressed.

- A custom Android application was developed to ensure proper data management and proper connection with the sensors and information obtained from the wireless sensors (Figure 3). The application used some software components (to handle Bluetooth and the management of the data) of an open mobile platform [28].
- The application had a 3-second countdown to ensure that no data was lost and a warning sound to signal the end of monitoring (second picture of Figure 3).
- In order to be useful for future studies, the application specifies the physical activity to be performed (watching TV, walking, sweeping the floor, *etc*.), the location of the smartphone (chest, leg, arm, *etc*.) and the user’s age group (young person, adult, elderly person, *etc*.) (first picture in Figure 3). For the experiments conducted in this study, the second and third options were always the hip and young person, respectively. These options do not affect data collection and are only used so that the csv files can easily be classified with the raw data.
- Each raw data unit (x,y,z) from the different accelerometers has a timestamp to avoid duplicating data for transmission problems.
- Custom desktop-software was developed in order to process all the raw data obtained by the Android application. This software implements the EE estimation procedure described in Section 4.3.
- All the experiments were conducted with the devices worn on the same part of the hip.

## 5. Results and Discussion

This section displays the results of the experiments conducted (Table 2, Table 3 and Table 4).

**Table 2:** Summary of the EE for the physical activity “Walking at 4 km/h”.

Walking 4 km/h	Subject 1		Subject 2		Subject 3		Subject 4		Subject 5	
	Count	MET	Count	MET	Count	MET	Count	MET	Count	MET
Nexus	2624	3.27	2443	3.15	2199	2.99	2126	2.94	1913	2.8
Samsung	455	1.84	508	1.88	392	1.80	398	1.80	365	1.78
LG L9	1350	2.43	2722	3.33	2728	3.34	2687	3.31	2243	3.02
Zephyr	6190	5.61	8632	7.22	6524	5.83	5936	5.45	5643	5.25
SensorTag	343	1.77	225	1.69	189	1.67	170	1.65	186	1.66

**Table 3:** Summary of the EE for the physical activity “Sweeping the floor”.

Sweeping the Floor	Subject 1			Subject 2		Subject 3		Subject 4		Subject 5	
	Count		MET	Count	MET	Count	MET	Count	MET	Count	MET
Nexus	1323	2.41		1034	2.22	1230	2.35	838	2.09	882	2.12
Samsung	376	1.79		325	1.76	413	1.81	282	1.73	360	1.78
LG L9	1516	2.54		1154	2.30	1149	2.30	833	2.09	1069	2.25
Zephyr	3719	3.99		1711	2.67	2718	3.33	2129	2.94	2315	3.06
SensorTag	121	1.62		95	1.60	118	1.62	93	1.60	122	1.62

**Table 4:** Summary of the EE for the physical activity “Watching TV”.

Watching TV	Subject 1			Subject 2		Subject 3		Subject 4		Subject 5	
	Count		MET	Count	MET	Count	MET	Count	MET	Count	MET
Nexus 5	126	1.62		112	1.62	102	1.61	143	1.64	105	1.61
Samsung	58	1.58		48	1.57	48	1.57	60	1.58	56	1.58
LG	155	1.64		151	1.64	152	1.64	178	1.66	142	1.64
Zephyr	1381	2.45		720	2.02	208	1.68	883	2.12	1641	2.62
SensorTag	43	1.57		40	1.57	42	1.57	44	1.57	41	1.57

Since the goal of the study was to determine how the device features affected EE estimation and to attempt to clarify the correlation between the features and results, the study of the results focuses on the devices and activities rather than user characteristics. Accordingly, the following charts show different disseminations obtained from the raw data management.

The different results and charts show different accelerometers providing a different number of counts and METs in the same activities performed by the same subjects. Since all the devices were used in the same place and each device recorded exactly the same exercise being carried out by the same subject over the same period of time, the source of such variations must lie in the specific features of each device. This is the case of acceleration sensitivity, which for the devices used for the experiment ranged considerably from ±2 g of SensorTag to ±16 g for the other devices. This meant that for accelerations above or below these thresholds, the sensors would not be able to measure accurate data. More particularly, ±16 g seems to be appropriate precision for most physical activities, such as the ones used in this study. The opposite happens with SensorTag, which can only detect accelerations of up to ±2 g, and is therefore incapable of measuring out-of-range accelerations, which seems to be inadequate. SensorTag is currently the device that on average provides the least accuracy for theoretical EE estimations.

**Figure 4:**
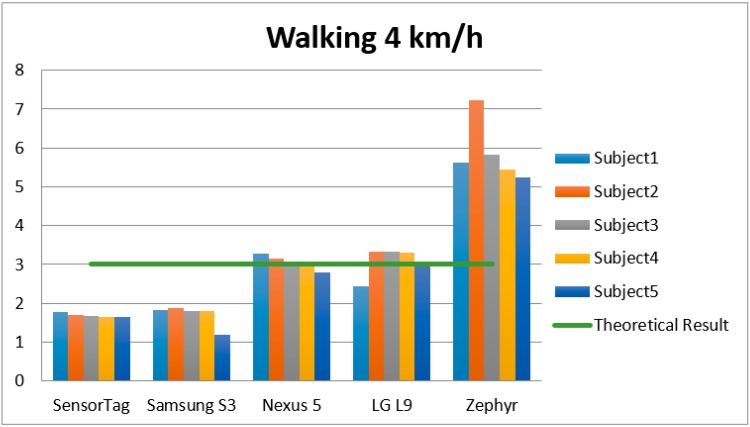
EE of the different accelerometers and subjects for the PA “Walking at 4 km/h”.

**Figure 5:**
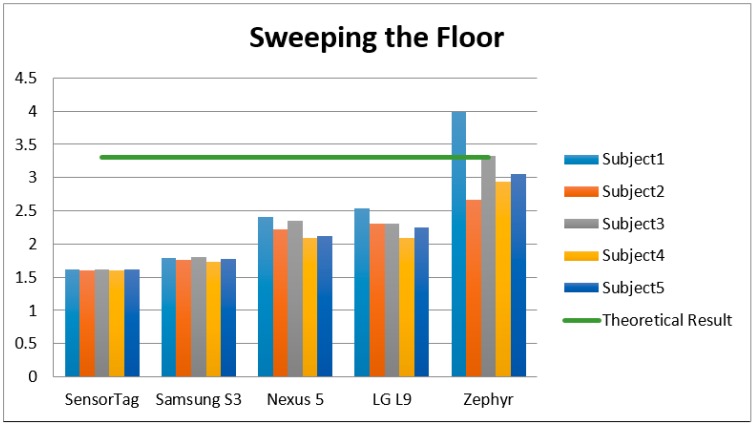
EE of the different accelerometers and subjects for the PA “Sweeping the floor”.

**Figure 6:**
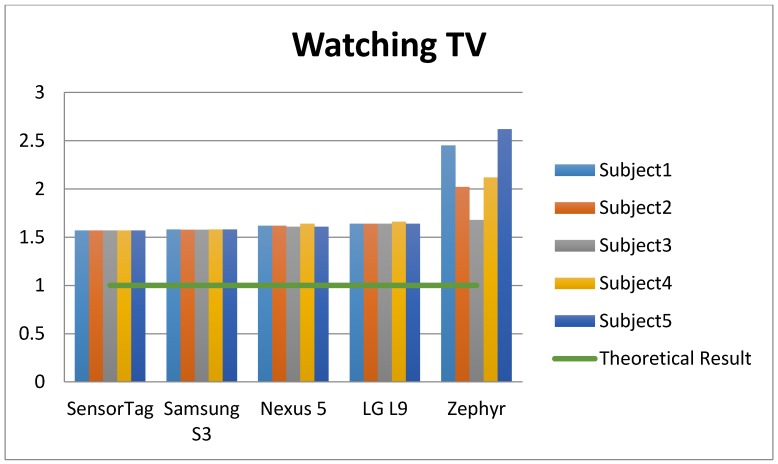
EE of the different accelerometers and subjects for the PA “Watching TV”.

According to the summary Table 1 of the study and Figure 4, Figure 5 and Figure 6, the sampling frequency of the device is determinant to compute the number of counts with the formulae used, and hence, the level of physical activity. According to Figure 4, Figure 5 and Figure 6, devices with a higher frequency obtain a higher number of counts, and consequently a higher number of METs, causing that even devices with a high frequency rate may overestimate the amount of the physical activity, such as the Zephyr in “Watching TV” activity.

In order to interpret the accuracy of calculi and measurements, we compared the results obtained in our study with one of the most referenced work in the area [5] in order to determine which accelerometers provided the nearest estimation to the theoretical reference measure. The referenced paper provided the measurement in METs for different activities, so we used this information to compare the results and determine which devices provided the nearest approximation with respect to the theoretical EE.

According to [5], the theoretical EE of “walking at 4 km/h” for 60 s is 3 METs. Figure 4 shows how the best estimations were provided by Smartphones 1 and 3 (Nexus and LG). With frequencies of 15 Hz and 20 Hz, these devices provided the nearest estimations to the theoretical METs. In the same way, for the activity “sweeping the floor”, the nearest estimation to the theoretical one was provided by the Zephyr device (3.3 METs was the real EE). In the final activity “watching TV”, none of the devices were close to 1 MET (the theoretical measurement), but the lowest margins of error were obtained by the non-smartphone accelerometer SensorTag (1 Hz).

Furthermore, our findings align and are coherent with previous experiments analyzing the performance of two popular accelerometers in an artificial, pure laboratory environment, *i.e*., without testing with real subjects and carrying out daily live activities [29].

The METs assessed with the different sensors reported lower values compared with the theoretical formula in PA (watching TV and walking) and higher baseline values (watching TV) [5]. These results showed that the [26] equation for calculating the AUC and the [27] equations for converting the counts to METs were not particularly accurate when calculating the EE of daily activities. The technique for calculating counts and METs is critical for predicting EE.

According to these results, the first conclusion is that the accelerometer features (frequency and G-force), as well as the technique for calculating the counts and METs, are crucial for obtaining a good EE estimation. Depending on the device features and the formulas used, in some activities the results obtained from certain devices were close to the theoretical results of the research presented in [5]. Changing the devices (or their features) or comparing the results with other reference pieces of work will also result in comparisons being different, different results being obtained and a different interpretation of whether the EE estimation is successful or not. Since this will always be present and the results will always be conditional, the possibility of adjusting this process to different requirements or parameters (e.g., the physical activity, device features or the user’s physiological information) might very well be the best approach for finding a solution to provide the most accurate EE estimation.

**Figure 7:**
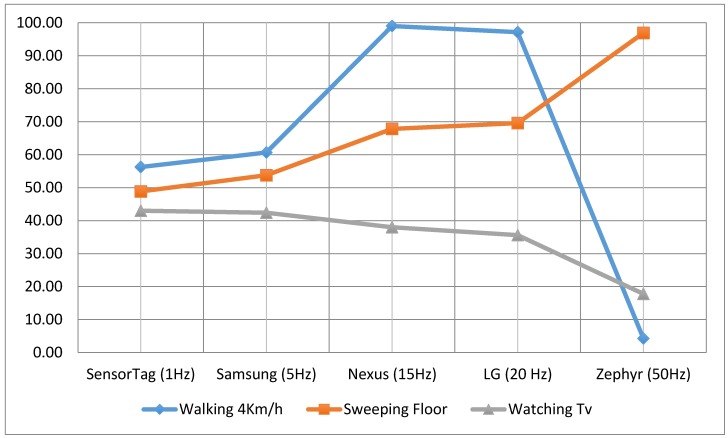
Relation between frequency (X axis) and accuracy of the results (Y axis).

To show how the accelerometer features influence the results, Figure 7 shows the relation between frequency (Hz) and the accuracy (relation between the experimental results and the theoretical results [5]). Each sample/slot shows the accuracy of the three activities for the calculated average of the five subjects, that is:

(3) $$\omega_{Activity}=\frac{\left(\frac{\sum_{i=1}^{5}\omega_{Activity\;\left(i\right)}}{5}\right)\times 100}{{Τ}_{Activity}}$$

where $\omega_{Activity\;\left(i\right)}$ is EE (in METS) of each subject and ${Τ}_{Activity}$ is the theoretical result for the activity processed [5].

The OS used for application development (Android) enabled the frequency of the accelerometer to be changed. For this study we used the default frequency for each smartphone (Table 1), but the correct adjustment of the frequency by varying formula parameters according to physiological characteristics (e.g., age, gender, weight, height) or even the use of more sophisticated solutions (e.g., expert systems which infer the appropriate frequency according to a learning system) could provide a promising solution in terms of usability, costs and results for estimating the EE.

In the first study [24] we focused on where the accelerometer was placed and we determined that the pocket was the best option. In this study we focused our experiments on the different device types and we were reasonably able to conclude that the device features, e.g., sampling rate and acceleration sensitivity, are crucial for obtaining a good EE estimation. Based on these results, future studies will be able to analyze different groups of people according to gender, age, height, weight and other physiological characteristics that could be decisive for finally determining how to estimate real EE according to the physical activity, user and device features

## 6. Conclusions

The estimation of energy expenditure (EE) in terms of metabolic equivalent tasks (METs) is crucial in certain contexts for enhancing the quality of life. Nowadays, one of most used techniques for estimating EE is the use of inertial devices, such as accelerometers, which are capable not only of measuring the “amount of movement” (quantified in count units) but also of objectively estimating EE (number of METs) using additional information.

In previous research we conducted various experiments to determine how differences between devices, physical activities and device location affected EE estimation. On the basis of that study, in the study presented in this paper various experiments were conducted to reinforce the previous results in order to determine the key elements that affect EE estimation.

These experiments have been performed by considering various requirements to ensure result reliability. In order to achieve this, the subjects performed the physical activities in the same way and custom support software was developed (e.g., an Android application and desktop software) to ensure proper data collection (no data loss and data coherence).

As our results show, the main conclusion of our study was that EE estimation based on count calculus using mobile devices highly depends on each device and still requires individualized correction techniques for each device. In other words, the technical device features (such as sampling frequency and acceleration sensitivity), the procedures used to estimate the amount of PA (counts) and the number of METs, as well as the referenced values for comparing the results, were crucial for determining the best EE estimation. Different smartphones with different accelerometers but with the same G-force sensitivity and a similar range of sampling frequencies provided similar results for the same activities. Our results showed that a higher frequency did not mean better EE estimation.

This conclusion has an important consequence since it will not be possible to estimate EE simply by using mobile devices in standalone fashion until the correction factor for each device and for each activity is determined. It is therefore difficult to extrapolate previously published results when different devices from those employed in the reference experiments are used. This hinders the applicability of EE estimation using mobile devices based on count calculus as the experiments must be conducted with identical devices each time or the results will be different and inconclusive. It also prevents the popularization of mobile, unsupervised EE analysis from becoming a reality in approaches such as those based on bring-your-own-device (BYOD) settings, since each person would bring a different device and the approach would not therefore be applicable.

By way of future work we will broaden the study to use a variety of smartphones placed on the hip to perform various activities with a larger sample of subjects grouped according to their physiological characteristics (e.g., age, gender, weight, height, health, *etc.*) in order to determine the correlation between accelerometer features, physical activity and the subject’s physiology. The definition of this correlation might well be crucial for developing future software adaptable solutions to enable the best EE approximation to be determined automatically irrespective of device features or other dependent parameters.

Likewise, we plan to repeat tests using accelerometers from the most widely referenced brand, *i.e*., ActiGraph, so as to find out if it is possible to define correction factors across different devices and provide a method to estimate energy expenditure with acceptable margins of errors.
